# Efflux ABC transporters in drug disposition and their posttranscriptional gene regulation by microRNAs

**DOI:** 10.3389/fphar.2024.1423416

**Published:** 2024-07-24

**Authors:** Yimei Wang, Mei-Juan Tu, Ai-Ming Yu

**Affiliations:** Department of Biochemistry and Molecular Medicine, School of Medicine, University of California at Davis, Sacramento, CA, United States

**Keywords:** ABC transporter, gene regulation, multidrug resistance, microRNA, ADME, pharmacokinetics, cancer, disease

## Abstract

ATP-binding cassette (ABC) transporters are transmembrane proteins expressed commonly in metabolic and excretory organs to control xenobiotic or endobiotic disposition and maintain their homeostasis. Changes in ABC transporter expression may directly affect the pharmacokinetics of relevant drugs involving absorption, distribution, metabolism, and excretion (ADME) processes. Indeed, overexpression of efflux ABC transporters in cancer cells or bacteria limits drug exposure and causes therapeutic failure that is known as multidrug resistance (MDR). With the discovery of functional noncoding microRNAs (miRNAs) produced from the genome, many miRNAs have been revealed to govern posttranscriptional gene regulation of ABC transporters, which shall improve our understanding of complex mechanism behind the overexpression of ABC transporters linked to MDR. In this article, we first overview the expression and localization of important ABC transporters in human tissues and their clinical importance regarding ADME as well as MDR. Further, we summarize miRNA-controlled posttranscriptional gene regulation of ABC transporters and effects on ADME and MDR. Additionally, we discuss the development and utilization of novel bioengineered miRNA agents to modulate ABC transporter gene expression and subsequent influence on cellular drug accumulation and chemosensitivity. Findings on posttranscriptional gene regulation of ABC transporters shall not only improve our understanding of mechanisms behind variable ADME but also provide insight into developing new means towards rational and more effective pharmacotherapies.

## 1 Introduction

ATP-binding cassette (ABC) transporters are a superfamily of transmembrane proteins which transport substrates by overcoming the concentration gradients across the membrane critical for cell functions (Robey et al., 2018; Wang et al., 2021). ABC transporters are expressed ubiquitously in human body while they are relatively more extensively distributed in metabolic organs such as small intestine and liver (Robey et al., 2018; Koehn, 2021). When ABC transporter domains recognize respective substrates, including xenobiotics (e.g., anticancer drugs and environmental agents) and endogenous compounds (e.g., nutrients and hormones), these transporters dispose such agents to maintain cell functions (Wang et al., 2021).

The clinical significance of ABC transporters has been well recognized. Since ABC transporters contribute to the homeostasis of some nutrients while exporting toxins, genetic mutations of ABC transporters might change transporter functions and be involved in certain disorders such as cystic fibrosis and retinal degeneration (Dean, 2005). On the other hand, the roles of ABC transporters in drug absorption, distribution, metabolism, and excretion (ADME) or pharmacokinetics (PK) are firmly established (Choi and Yu, 2014; Koehn, 2021), and overexpression of efflux ABC transporters in carcinoma cells confers multidrug resistance (MDR) (Choi and Yu, 2014; Bukowski et al., 2020; Koehn, 2021). Moreover, more than 90% of cancer patient deaths are caused by MDR during chemotherapy (Bukowski et al., 2020), in which the overexpression of efflux ABC transporters is considered to be one of the most important mechanisms (Choi and Yu, 2014; Fan et al., 2023). Therefore, understanding the factors in the control of ABC efflux transporter gene expression is important for identifying proper ways to overcome the MDR towards an improved therapy, in addition to the development of chemical inhibitors.

Studies have revealed the importance of several nuclear receptors, such as pregnane X receptor (PXR), constitutive androstane receptor (CAR), proliferator-activated receptor (PPAR) and aryl hydrocarbon receptor (AHR), in transcriptional gene expression of ABC transporters (Chen et al., 2012). For instance, PXR has been shown to regulate ABCB1 expression in human colon cancer LS174T cells through direct binding to the direct repeat separated by four base pairs (DR4) motif within the ABCB1 promoter region, and thus PXR ligand rifampin induces ABCB1 expression (Geick et al., 2001). As another example, 3-methylcholanthrene (3MC) upregulates ABCG2 expression in colon cancer LS174T cells via the activation of AHR which binds to the AHR response element 5 within ABCG2 promoter region and thus activate the transcription of ABCG2 (Tompkins et al., 2010).

Recent research has also revealed posttranscriptional gene regulation (PTGR) of ABC transporters by the genome-derived, small noncoding microRNAs (miRNAs or miRs) (Yu and Pan, 2012; Ingelman-Sundberg et al., 2013; Zhong and Leeder, 2013; Yu et al., 2016). By acting on the 3′-untranslated region (3′UTR) of target transcripts, miRNAs lead to the inhibition of mRNA translation or enhancement of mRNA degradation (Lai, 2002; Ambros, 2004). For example, hsa-miR-519c has been shown to target the 3′UTR of ABCG2 to modulate its protein outcomes in parental human colon cancer S1 cells whereas not in the drug-resistant S1MI80 cells, as the latter are comprised of shortened ABCG2 mRNA escaping miR-519c-controlled PTGR and leading to ABCG2 overexpression in the drug-resistant cells (To et al., 2008; To et al., 2009). In addition, hsa-miR-328 controls the PTGR of ABCG2 in the drug-resistant breast cancer cells to modulate mitoxantrone sensitivity (Pan et al., 2009). These findings not only offer insights into the presence of PTGR mechanisms behind variable ABC transporter expression levels and thus drug disposition capacities but also the development of new means to improve therapies.

Nevertheless, studies on miRNA-controlled PTGR have been limited to using vector or virus based expression materials or chemically synthesized miRNA agents (Yu et al., 2019; Traber and Yu, 2023). The former are not RNA but DNA molecules, while the latter are comprised of extensive artificial modifications differing from natural miRNAs. Therefore, novel recombinant technologies have been developed to achieve *in vivo* production of true biological miRNA agents, namely BERAs or BioRNAs (Li et al., 2014; Chen et al., 2015; Ho et al., 2018; Li et al., 2021; Tu et al., 2021; Traber et al., 2024). Recombinant miRNAs have been shown to be active and selectively regulate target gene expression, including many ABC transporters (Li et al., 2019; Yi et al., 2020), representing a new class of RNA molecules for basic research and showing potential as therapeutics (Ho et al., 2018; Cronin and Yu, 2023; Traber and Yu, 2023).

In this article, we first summarize the clinical importance of ABC transporters, specifically in ADME and MDR. Following a brief introduction of PTGR mechanism controlled by the genome-derived miRNAs, we overview the regulation of some important efflux ABC transporters by specific miRNAs and the consequent effects on drug transport and chemosensitivity. Further, we summarize the production and utilization of unparalleled recombinant miRNAs for the investigation of ABC transporter PTGR and discuss their potential applications.

## 2 Clinical importance of ABC transporters

### 2.1 Discovery and general properties of ABC transporters

Research on ABC transporters emerged following the discovery of a nutrient-transporting protein from membrane vesicles of *Escherichia coli* that was dependent on ATP hydrolysis (Berger and Heppel, 1974). Later, Juliano and Ling (1976) disclosed a 170-kD, surface membrane glycoprotein affecting colchicine permeation, namely P-glycoprotein (P-gp or ABCB1), which was responsible for drug resistance in the mammalian cells. Further genetic analyses of the mammalian and bacterial transporters revealed that their coding genes were highly conserved (Walker et al., 1982; Riordan et al., 1985). With valuable insights of human genome, many human ABC transporter genes were identified (Luciani et al., 1994; Allikmets et al., 1996; Klein et al., 1999; Dean et al., 2001; Olsen et al., 2001; Venter et al., 2001). A total of 48 ABC genes have been revealed to encode functional ABC transmembrane proteins in humans and classified into seven subfamilies, from ABCA to ABCG (Dean et al., 2001; Dean, 2005; Vasiliou et al., 2009; Alam and Locher, 2023).

**FIGURE 1:**
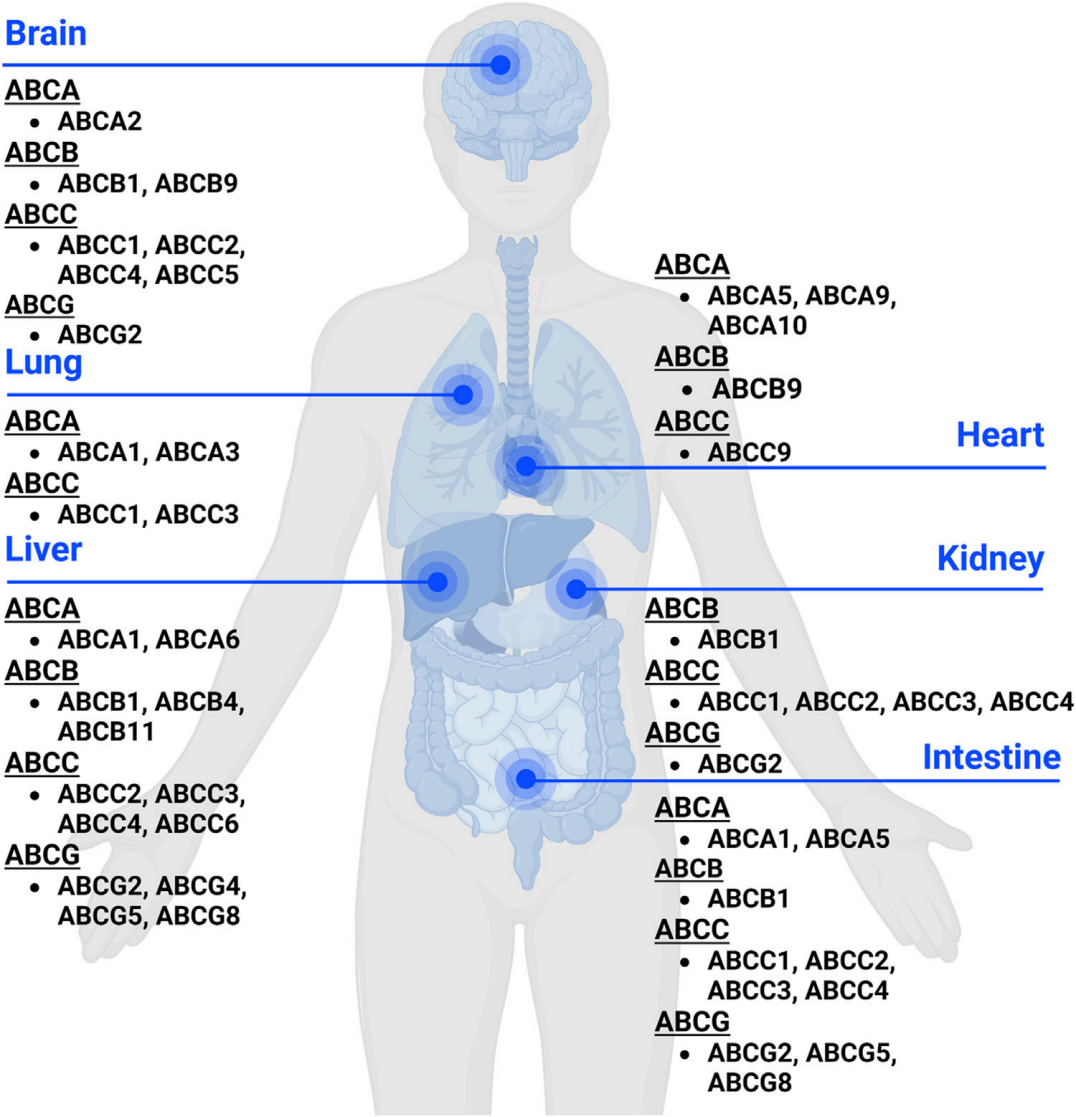
Expression of ABC transporters in human body. ABC transporters are mostly expressed in metabolic and excretory organs, such as liver, kidney, and intestine. This figure was created with BioRender.com.

**FIGURE 2:**
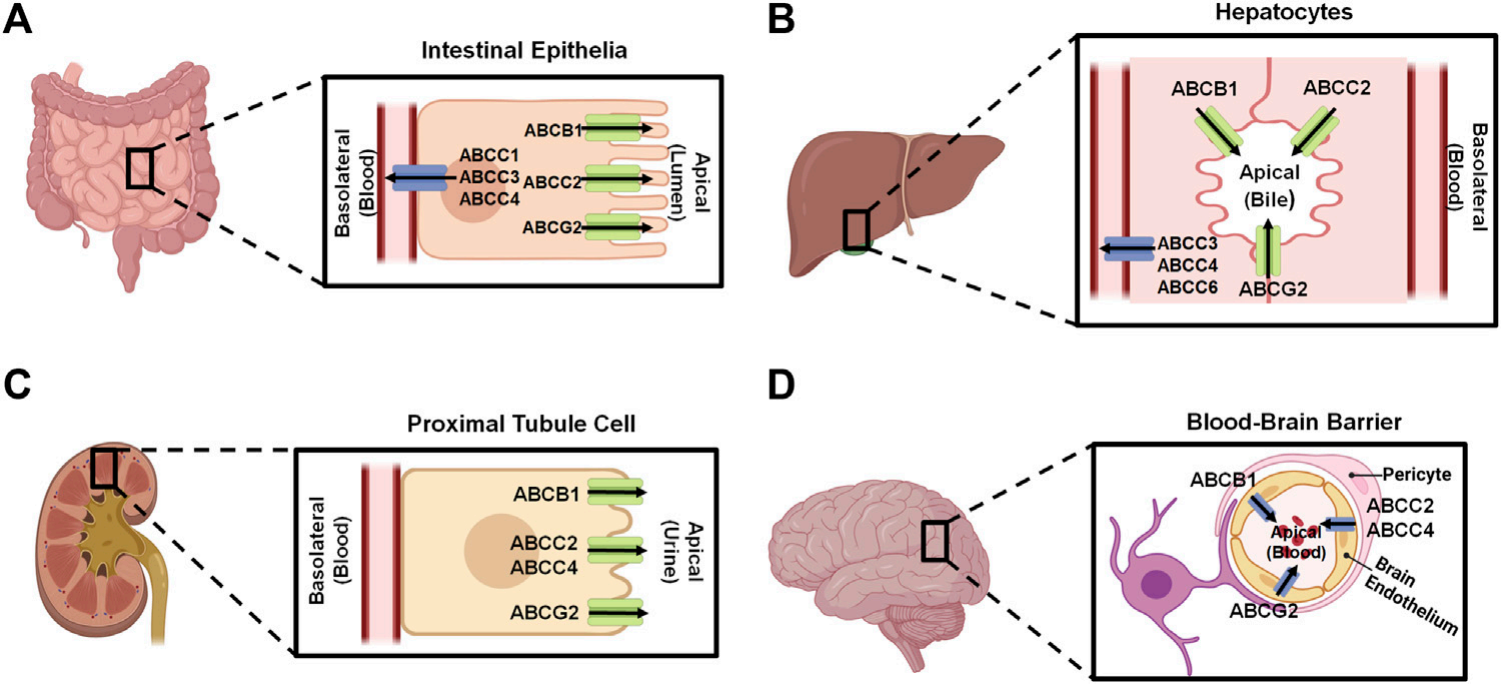
Localization of important ABC transporters in human intestine **(A)**, liver **(B)**, kidney **(C)**, and brain **(D)**. The apical side refers to the cell surface oriented towards the lumen, while the basolateral side facing the extracellular fluid, such as blood. ABC transporters located to the apical surfaces export substances from the tissues to the lumens, and transporters expressed at the basolateral surfaces facing the blood vessels substrate efflux from the tissues to the portal bloods. At the blood-brain barrier, transporters localized on the apical sides of brain endothelium cells facing the bloodstream transfer substrates into the blood. This figure was made by using BioRender.com.

Human ABC transporters are expressed in almost all organs (Figure 1), especially those metabolic tissues such as intestine, liver, and kidney to greater degrees. These transporters are also critical for the body homeostasis and physiology by translocating xenobiotics and endobiotics across cells, and defects of ABC transporters may contribute to certain types of metabolic diseases. For instance, ABCA1 is a membrane protein which maintains lipid homeostasis by effluxing phospholipids and cholesterol to the extracellular lipid-poor apolipoprotein (Dean et al., 2001; Vincent et al., 2019), and ABCA1 within adipose tissues contributes to the high-density lipoprotein (HDL) biogenesis in the body (Chung et al., 2011). Through analyzing the visceral and subcutaneous adipose tissue ABCA1 expression in both lean and obese individuals, Vincent et al. (2019) found that lower expression of ABCA1 was correlated with obesity and insulin resistance. Notably, the precise location of important ABC transporters (e.g., ABCB1, ABCC1-4, and ABCG2) in major organs relevant to ADME, including apical (cell faces to the lumen) and basolateral (faces to the extracellular fluid) sides of cells (Figure 2), have been well established for improved understanding of the directions of drug transport within the body.

An ABC transporter is usually comprised of two nucleotide-binding domains (NBDs) to which ATP is bound and two transmembrane domains (TMDs) that allow transporter protein to reside on cell membrane and interact with specific substrates (Dean et al., 2001; Choi and Yu, 2014; Fan et al., 2023). Indeed, the classification of the ABC transporters is based on the sequences of conserved NBDs consisting of characteristic motifs (Walker A and Walker B) and the ABC signature motif (Walker et al., 1982; Hyde et al., 1990; Dean et al., 2022). X-ray crystallography studies have showed a similar conformational change of ABCB1 transporters from non-human species upon interacting with poly-substrates (Jin et al., 2012; Esser et al., 2017). Most recent applications of novel cryo-electron microscopy (cryoEM) technology have provided further structures of ABC transporters (Kim and Chen, 2018; Nosol et al., 2020), including human ABCB1 in the outward-facing conformation as well as interactions with substrates and inhibitors. The latter shall facilitate the prediction of possible, transporter-based drug-drug interactions as well as the discovery and development of more effective inhibitors to overcome MDR.

### 2.2 Roles of ABC transporters in ADME

ABC transporters expressed in human body (Figures 1, 2) are known to directly affect drug absorption, distribution, and excretion as well as metabolism via interacting with metabolic enzymes. Among various factors affecting oral drug absorption from gastrointestinal tract to the bloodstream (Stillhart et al., 2020), transporters ABCB1, ABCC2, and ABCG2 localized on the apical sides of intestinal epithelial cells (Figure 2A) contribute to limiting oral absorption while ABCC1, ABCC3, and ABCC4 on the basolateral sides facilitate the absorption of substrate drugs. Digoxin, a cardiac glycoside used to enhance heart function, has been identified as one of many substrates of ABCB1 (Table 1), and the absorption and overall PK of digoxin may be determined by ABCB1 status (de Lannoy and Silverman, 1992; Fromm et al., 1999; Greiner et al., 1999). Indeed, ABCB1 inducer rifampin was shown to have significant impact on the PK of digoxin in healthy volunteers, associated with the increase in intestinal ABCB1 expression levels (Greiner et al., 1999). Besides clinical investigations, genetically modified mouse models are widely used to define the importance of ABC transporters in ADME/PK (Jiang et al., 2011; Durmus et al., 2015), including the role of ABCB1 in digoxin PK (Fromm et al., 1999). As another example, comparative studies with wild type, Abcc2-knockout, Abcb1a/1b-knockout, and Abcb1a/1b/Abcc2-knockout mice demonstrated the important role of Abcc2 and Abcb1 in paclitaxel PK, in particular, Abcc2 in hepatobiliary excretion (Figure 2B) over renal excretion (Figure 2C), as well as Abcb1 in intestinal absorption (Lagas et al., 2006).

**TABLE 1:** Localizations and common substrates of major human ABC transporters.

Transporter	Localization	Substrates
ABCB1	Extensively expressed in metabolic and excretory organs, including liver, intestine, and kidney; Located in the apical membrane (Thiebaut et al., 1987)	1. Anthracyclines Doxorubicin (Mechetner et al., 1998)
		2. Taxanes Paclitaxel (Mechetner et al., 1998), docetaxel (Shirakawa et al., 1999)
		3. Vinca alkaloids Vinblastine (Ueda et al., 1987), vincristine (Meyers et al., 1991)
		4. Cyclin-dependent kinase inhibitors Palbociclib (De Gooijer et al., 2015), ribociclib (Sorf et al., 2018; Martínez-Chávez et al., 2019)
		5. Tyrosine kinase inhibitors Imatinib (Hegedűs et al., 2002), nilotinib (Tiwari et al., 2009), dasatinib (Chen et al., 2009), gefitinib (Kitazaki et al., 2005)
		6. Cardiac glycoside Digoxin (de Lannoy and Silverman, 1992)
ABCC1	Ubiquitously expressed in human organs; Located in the basolateral membrane (Evers et al., 1996; Flens et al., 1996)	1. HIV protease inhibitors Saquinavir (Williams et al., 2002), ritonavir (Meaden et al., 2002), lopinavir (Janneh et al., 2007)
		2. Antimetabolites Methotrexate (Zeng et al., 2001), edatrexate (Zeng et al., 2001)
		3. Anthracyclines Doxorubicin (Cole et al., 1992), daunorubicin (Renes et al., 1999), epirubicin (Davey et al., 1995)
		4. Topoisomerase inhibitors Etoposide (Lorico et al., 1997), irinotecan (Chu et al., 1999)
		5. Statins Rosuvastatin (Knauer et al., 2010), atorvastatin (Knauer et al., 2010)
		6. Tyrosine kinase inhibitors Imatinib (Hegedűs et al., 2002)
ABCC2	Mostly expressed in liver, intestine, and kidney; located in the apical membrane (Keppler and König, 1997; Jedlitschky et al., 2006)	1. Antibiotics Grepafloxacin (Naruhashi et al., 2002)
		2. HIV protease inhibitors Ritonavir (Huisman et al., 2002), indinavir (Huisman et al., 2002), saquinavir (Su et al., 2004)
		3. Antimetabolites Methotrexate (Chen et al., 2003)
		4. Alkylating antineoplastic agent Cisplatin (Cui et al., 1999)
		5. Anthracyclines Doxorubicin (Cui et al., 1999), epirubicin (Cui et al., 1999)
		6. Topoisomerase inhibitors Etoposide (Cui et al., 1999)
ABCC3	Mainly expressed in liver and located in the basolateral membrane (Kool et al., 1999)	1. Antimetabolites Methotrexate (Kool et al., 1999)
		2. Vinca alkaloids Vincristine (Zeng et al., 1999)
		3. Topoisomerase inhibitors Teniposide (Kool et al., 1999), etoposide (Kool et al., 1999)
ABCC4	Expressed in liver and kidney; Located in the basolateral membrane of liver (Rius et al., 2003), and in the apical membrane of kidney (van Aubel et al., 2002)	1. Antimetabolites Methotrexate (van Aubel et al., 2002), 6-mercaptopurine (Chen et al., 2001)
		2. Topoisomerase inhibitors Topotecan (Leggas et al., 2004), irinotecan (Norris et al., 2005)
		3. Antivirals Nelfinavir (Fukuda et al., 2013), adefovir (Imaoka et al., 2007), tenofovir (Imaoka et al., 2007)
		4. Statins Rosuvastatin (Knauer et al., 2010), atorvastatin (Knauer et al., 2010)
ABCC5	Highly expressed in brain and muscle; Located in the basolateral membranes of most cells (Wijnholds et al., 2000), but in the apical membrane of brain endothelial cells (Zhang et al., 2004)	1. Nucleobase analogs 6-Mercaptopurine (Wijnholds et al., 2000), thioguanine (Wijnholds et al., 2000)
		2. Topoisomerase inhibitors Irinotecan (Di Martino et al., 2011)
		3. Antimetabolites 5-FU (Wijnholds et al., 2000; Pratt et al., 2005), pemetrexed (Chen et al., 2021)
		4. Statins Rosuvastatin (Knauer et al., 2010), atorvastatin (Knauer et al., 2010)
ABCC10	Mostly expressed in liver, brain, and colon; Located in the basolateral membrane (Hopper-Borge et al., 2004)	1. Taxanes Docetaxel (Hopper-Borge et al., 2004), paclitaxel (Hopper-Borge et al., 2004)
		2. Vinca alkaloids Vinblastine (Hopper-Borge et al., 2004), vincristine (Hopper-Borge et al., 2004)
		3. Tyrosine kinase inhibitors Imatinib (Shen et al., 2009), nilotinib (Shen et al., 2009)
ABCG2	Highly expressed in liver, kidney, and intestine; Located in the apical membrane (Rocchi et al., 2000)	1. Topoisomerase inhibitors Mitoxantrone (Rocchi et al., 2000), topotecan (Rocchi et al., 2000)
		2. Tyrosine kinase inhibitors Imatinib (Burger et al., 2004), gefitinib (Lemos et al., 2009), nilotinib (Hegedűs et al., 2009), dasatinib (Hegedűs et al., 2009)
		3. Cyclin-dependent kinase inhibitors Palbociclib (De Gooijer et al., 2015)
		4. Anthracyclines Daunorubicin (Robey et al., 2003), doxorubicin (Robey et al., 2003)

ABC transporters may restrict the distribution of substrate drugs (Table 1) from the bloodstream to target organs, thereby the study of how ABC transporters impede drug penetration into the central nervous system (CNS) remains an important area in ADME/PK or drug delivery fields (Daood et al., 2008; Miller, 2015). ABCB1 and ABCG2 are two major efflux transporters expressed at the apical sides of brain endothelium cells (Figure 2D) which prevent substrates across the blood-brain barrier (BBB) and protect the brain from toxins or other xenobiotics including many therapeutic drugs (Dean et al., 2001; Daood et al., 2008). For instance, cyclin-dependent kinase inhibitors (CDKis) (Table 1) are a group of medications approved for the treatment of metastatic breast cancer, and it is important to understand their penetration into the brain (De Gooijer et al., 2015; Martínez-Chávez et al., 2019). *In vitro* studies revealed the CDKi ribociclib as a substrate for human ABCB1 whereas poorly transported by mouse abcg2 (Sorf et al., 2018; Martínez-Chávez et al., 2019). *In vivo* study with abcb1 and abcg2 knockout mice not only showed a 2.3-fold increase of ribociclib oral bioavailability but also 30-fold sharp elevation of brain-to-plasma (K_p,brain_) ratio, supporting the role of abcb1 in brain distribution of ribociclib (Martínez-Chávez et al., 2019). Additionally, the coadministration of an abcb1 and abcg2 dual inhibitor (e.g., elacridar) resulted in a significant increase in ribociclib concentrations in the brain (Martínez-Chávez et al., 2019). Similarly, the other CDKi palbociclib exhibited higher brain distribution when abcb1 and abcg2 were knocked out in mice (De Gooijer et al., 2015).

Kidney is the primary excretory organ which eliminates drugs, metabolites, and toxins. Therefore, some efflux transporters (e.g., ABCC2, ABCC4, and ABCG2) located on the apical membranes of kidney proximal tubule cells (Figure 2C) may pump their substrates into the urine and contribute to their elimination (Masereeuw and Russel, 2012). Adefovir and tenofovir (Table 1), two antiviral drugs, were identified as ABCC4 substrates (Imaoka et al., 2007). Compared to wild-type mice, abcc4-knockout mice exhibited higher plasma drug concentrations and kidney accumulation following adefovir treatment. Additionally, the total clearance (CL_total_) of adefovir was found to be lower in the absence of abcc4, and tubular secretion clearance (CL_renal, kidney_) of adefovir and tenofovir was also reduced over 50% in the knockout mice (Imaoka et al., 2007).

Clinical investigations have further revealed an altered expression of ABC transporters among patients with acute or chronic diseases and potential influence on drug ADME, efficacy or toxicity (Evers et al., 2018; Laddha et al., 2024). One study on hyperbilirubinemia and association with bile acid homeostasis and regulation among intensive care unit patients showed that protein levels of ABCC3 (Figure 2B) were strongly increased at the basolateral sides of patient liver biopsies (Vanwijngaerden et al., 2011). Another study with intestinal biopsy specimens from healthy volunteers as well as human immunodeficiency virus (HIV)-positive subjects with or without antiretroviral therapy revealed a significantly lower mRNA and protein levels of ABCC2 in antiretroviral therapy-naive subjects than the control group which was partially restored to baseline levels along with significant increase in ABCB1 expression in HIV-positive subjects following antiretroviral therapy (Kis et al., 2016). A very recent article provides a comprehensive review on the expression and function of hepatic transporters under fatty liver conditions, as well as subsequent effects on the PK properties of relevant drugs, including some clinical findings (Laddha et al., 2024).

### 2.3 Roles of ABC transporters in MDR

**FIGURE 3:**
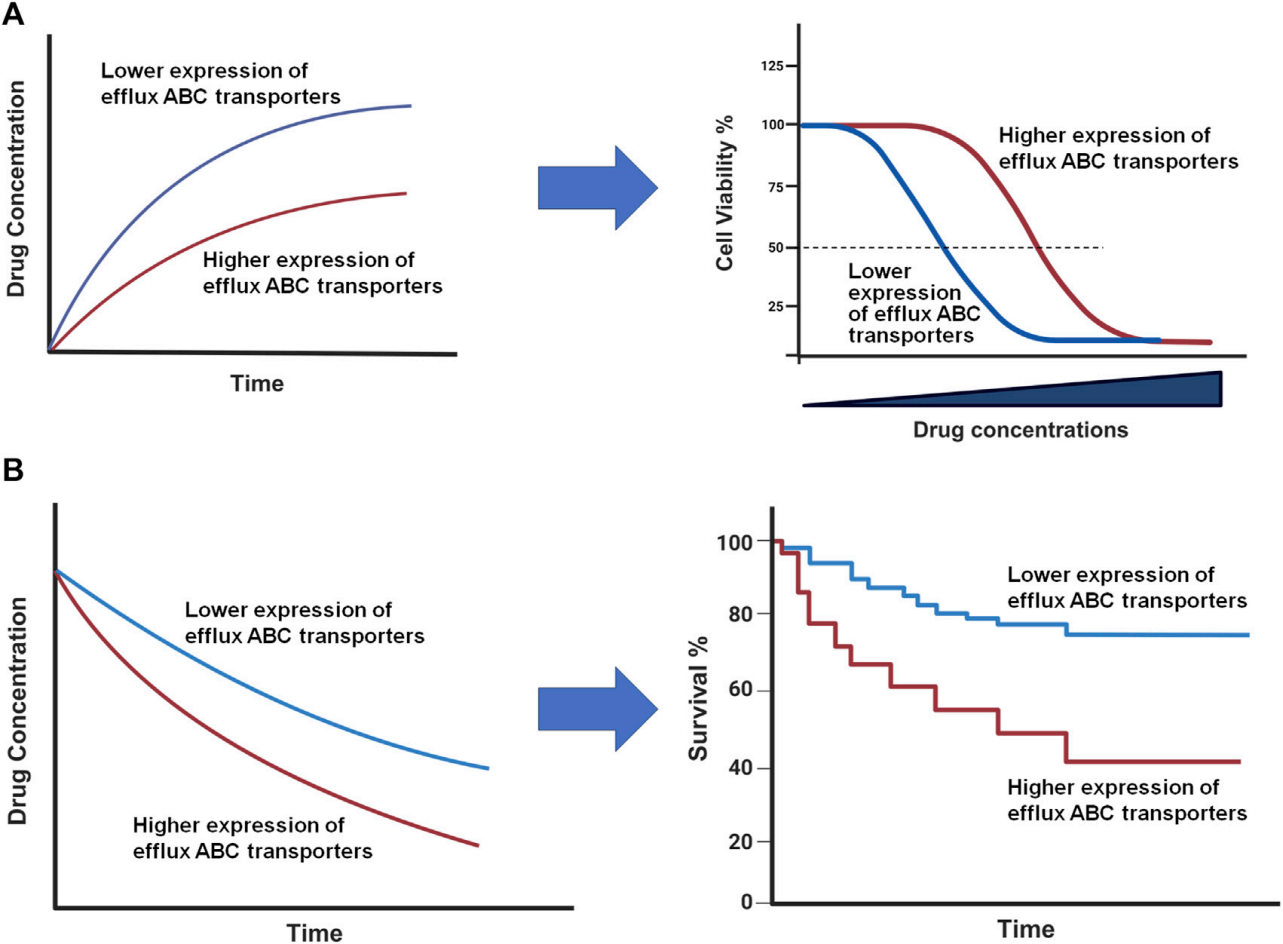
Impact of ABC transporters on drug exposure and therapeutic outcome *in vitro* **(A)** and *in vivo* **(B)**. **(A)** Overexpression of efflux ABC transporters leads to a lower intracellular drug accumulation (left) and antiproliferation activity (right). **(B)** Drug concentrations are reduced in patient tumor tissues with higher ABC transporter expression (left), which may lead to a lower survival rate (right). This figure was created with BioRender.com.

Besides their importance in controlling drug ADME/PK, the overexpression of efflux ABC transporters is a common mechanism behind MDR (Figure 3) (Hrycyna and Gottesman, 1998; Choi and Yu, 2014; Robey et al., 2018; Orelle et al., 2019) that leads to pharmacotherapy failure among patients with infections, cancers, or other diseases. Specific ABC transporters, including ABCB1, ABCC family, and ABCG2, have been shown to be overexpressed in human carcinoma cells to confer MDR, attributed to a lower intracellular drug exposure (Figure 3A) and manifested by low or lost chemosensitivity (Figure 3B). Among them, ABCB1 was highly expressed in almost all types of MDR cancers, such as adrenocortical, colon, breast, and kidney; and the contribution of ABCB1 to MDR has been proven for many drugs, including vincristine, doxorubicin (DOX), and 5-fluorouracil (5-FU) (Fojo et al., 1987) (Table 1). MDR appeared after the initial chemotherapy (e.g., vincristine) with the increase in ABCB1 levels during relapse and eventually led to mortality (Fojo et al., 1987). Another study also revealed much higher frequency or extent of ABCB1 expression among relapse acute nonlymphoblastic leukemia (ANLL) patients (60%) than initial ANLL patients (26%) (Han et al., 2001), indicating the involvement of efflux transporter ABCB1 in MDR.

ABCC family transporters were found to be highly expressed in lung cancers associated with MDR, including both small cell lung cancer (SCLC) and non-small cell lung cancer (NSCLC) (Young et al., 1999). Among them, ABCC3 was revealed to contribute greatly to the resistance to both DOX and vincristine (Table 1) during the treatment of lung cancer while other ABCC transporters could be involved in it. As another example, imatinib was identified as a substrate of ABCG2 (Table 1) (Burger et al., 2004) in treating leukemia, and ABCG2 was found to be upregulated in imatinib-resistant leukemia cells (Kaehler et al., 2017). Further, overexpression of ABCG2 was demonstrated to correlate with the resistance to many other drugs such as mitoxantrone (Rocchi et al., 2000), daunorubicin (Robey et al., 2003), DOX (Robey et al., 2003), and topotecan (Rocchi et al., 2000).

While overexpression of efflux ABC transporters confers MDR in carcinoma cells, it is also accompanied by many other cellular changes (Choi and Yu, 2014). For instance, MDR cells were revealed to exhibit a higher intracellular pH than parental cells (Boscoboinik et al., 1990), and the increased intracellular pH in MDR cells might decrease apoptosis as the caspase-dependent apoptosis was elevated under acidic conditions (Muriithi et al., 2020). Indeed, the reversal of MDR by small molecules were not necessarily correlated with their effects on intracellular pH (Boscoboinik et al., 1990). Furthermore, lipid compositions of plasma membranes were found to be altered in MDR cells, such as higher levels of sphingomyelin, phosphatidylinositol, cholesterol, cholesterol esters in MDR cell membranes (Peetla et al., 2013; Kopecka et al., 2020), which could contribute to the altered permeability of anticancer drugs (Mohammad et al., 2020; Szlasa et al., 2020).

As the impact of high-expressing efflux ABC transporters on the sensitivity of cells to respective substrate drugs, such as DOX (Fojo et al., 1987; Lemontt et al., 1988; Kim et al., 2015), has well demonstrated by preclinical studies (Figure 3A), clinical studies have also showed that high levels of ABC transporters contribute to the limited tissue and/or systemic drug exposure and subsequently poor therapeutic outcomes (Figure 3B) albeit there are some controversial reports (Tamaki et al., 2011). A recent study on the expression status of various ABC transporters in different tumors revealed that, while ABC transporter expression correlated with different stages of breast, kidney or lung tumors, a lower ABCB1 mRNA expression level was predictive of significantly longer survival of patients with ovarian or kidney cancer and thymoma (Kadioglu et al., 2020), supporting the clinical significance of efflux ABC transporters and needs for new means to achieve individualized pharmacotherapy.

One way to overcome MDR is to develop and co-administer with ABC transporter inhibitors (Robey et al., 2018), which rather has not found any success, given the complex roles of these transporters in ADME/PK (Choi and Yu, 2014). Understanding the mechanisms by which efflux transporters are overexpressed in MDR cells and organisms may offer clues for developing new strategies. Indeed, various factors such as gene duplication or multiplication as well as the changes in transcriptional regulation or signaling have been shown to contribute to the overexpression of efflux ABC transporters, and respective remedies may be explored and critically evaluated.

## 3 MicroRNAs in posttranscriptional gene regulation

MiRNAs are small noncoding RNAs generated from the genome, around 22 nt in length, which play important roles in PTGR in cells (Ambros, 2004). Lin-4 (Lee et al., 1993) and let-7 (Reinhart et al., 2000) are among the first functional miRNAs discovered in *Caenorhabditis elegans*, while let-7 is the first miRNA identified in humans (Pasquinelli et al., 2000). With more studies to the miRNAs, they have been found across many animal species which are highly conserved (Pasquinelli et al., 2000; Li et al., 2010; De Rie et al., 2017). Regulation of target genes by miRNAs loaded within the RNA-induced silencing complex (RISC) involve the inhibition of mRNA translation and enhancement of mRNA decay or degradation (Lee et al., 1993; Lai, 2002; Doench and Sharp, 2004). Through PTGR of targeted genes, miRNAs influence essentially all biological processes, including those important in maintaining metabolite homeostasis and cellular defenses (Ha and Kim, 2014). Furthermore, miRNAs hold promise as therapeutic targets or entities for the treatment of various human diseases (Yu et al., 2020; Yu and Tu, 2022; Traber and Yu, 2023, 2024).

### 3.1 MicroRNA biogenesis

**FIGURE 4:**
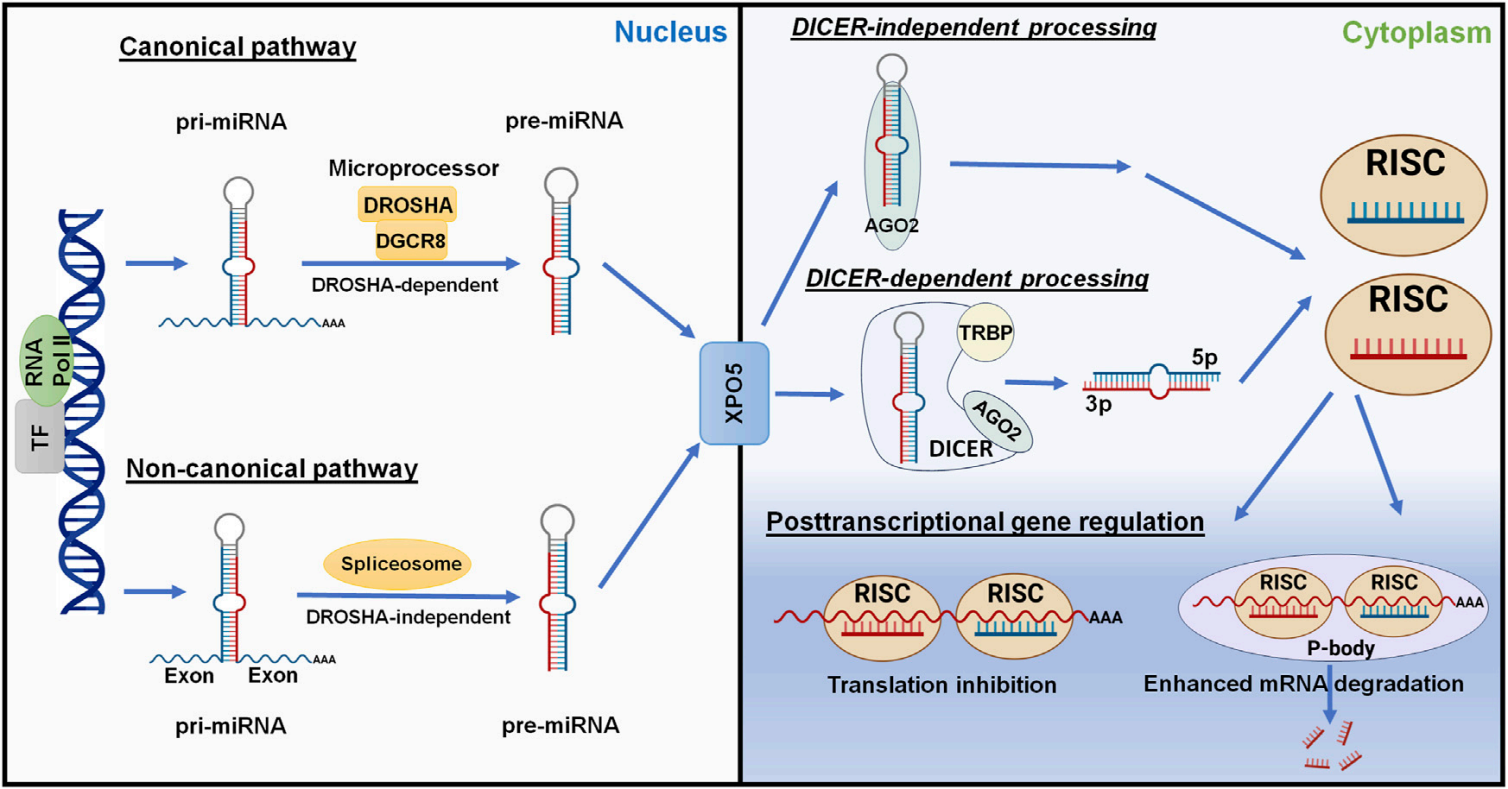
Posttranscriptional gene regulation controlled by the genome-derived miRNAs. The canonical and non-canonical pathways of miRNA biogenesis are dependent and independent upon DROSHA, respectively, to generate pre-miRNAs from longer pri-miRNAs transcribed from the genome within nucleus. Following Exportin-5 (XPO5)-mediated translocation, cytoplasmic pre-miRNAs are recognized and processed by DICER, TRBP, and AGO2 to the miRNA duplex. The AGO protein unwinds the miRNA duplex where either the 5p or 3p strand may be incorporated into the RISC. Some mature miRNAs are generated through DICER-independent processing as pre-miRNAs directly bind to AGO2. The mature miRNA usually acts on the 3′UTR of target mRNA, leading to translation inhibition or enhanced mRNA degradation. This figure was made by using BioRender.com and Microsoft Powerpoint.

Most miRNAs are transcribed from corresponding coding genes by RNA polymerase II (Pol II) (Lee et al., 2004) to primary miRNAs (pri-miRNAs) which are recognized and cleaved by micro-processor proteins, DROSHA and DGCR8, to generate short-hairpin, precursor miRNAs (pre-miRNAs) within the nucleus (Lee et al., 2003; Denli et al., 2004; Gregory et al., 2004; Wang et al., 2007) (Figure 4). After the pre-miRNA is translocated from nucleus to cytoplasm by Exportin-5 (XPO5) (Yi et al., 2003; Lund et al., 2004), it is further processed by the RISC-loading complex (RLC), consisting of the RNase III family protein DICER (Bernstein et al., 2001; Ketting et al., 2001), transactivation response element RNA-binding protein (TRBP) (Lee et al., 2003; Fareh et al., 2016), and Argonaute 2 (AGO2) protein (Liu et al., 2004; Chendrimada et al., 2005; MacRae et al., 2008), to miRNA duplex. Subsequently, the AGO protein unwinds the miRNA duplex and guides one strand (3p- or 5p-) into the RISC (Khvorova et al., 2003; Kwak and Tomari, 2012) to achieve RNA interference (RNAi) (Figure 4).

Complementary to the canonical biogenesis pathway, the maturation of some miRNAs is independent on the micro-processor (DROSHA/DGCR8) or DICER to offer noncanonical miRNAs (Figure 4). The short-hairpin introns may be spliced and debranched to produce pre-miRNAs directly (Okamura et al., 2007; Ruby et al., 2007). After exported into the cytoplasm, noncanonical pre-miRNAs may follow the DICER-dependent maturation pathway (Okamura et al., 2007). This DROSHA-independent mirtron pathway is conserved for some miRNAs (e.g., miR-887) including mammals (Berezikov et al., 2007; Babiarz et al., 2011). On the other hand, some miRNAs (e.g., miR-451) is formed independent on DICER (Figure 4), despite that pre-miRNA production is DROSHA-dependent. AGO2 protein has been revealed as an alternative enzyme to directly cleave pre-miRNA (Cheloufi et al., 2010; Yang et al., 2010), which may involve subsequent trimming by poly(A)-specific ribonuclease (PARN) (Yoda et al., 2013; Kim et al., 2016), to generate functional miRNA strand for target silencing within the RISC (Figure 4). Interestingly, miR-451 generally partners with miR-144, relying on the transfer of micro-processor proteins from miR-144 to continue its further maturation (Yang et al., 2010; Shang et al., 2020).

### 3.2 Posttranscriptional gene regulation by microRNAs

Derived from either canonical or noncanonical pathway, functional miRNA activates the RISC to control target gene expression at the posttranscriptional level, namely PTGR. In particular, one strand (5p- or 3p-) recognizes target mRNA sequences through imperfect base pairing to induce translation inhibition or mRNA cleavage or degradation (Figure 4). The human genome contains four AGO proteins (AGO1-4), among them AGO2 plays a major role in PTGR (Liu et al., 2004; O’Carroll et al., 2007). The seed region at the 5′ of an miRNA may include 7–8 nt that are fully complementary to the miRNA response element within 3′UTR of target mRNA to effectively inhibit mRNA translation and/or degradation (Doench and Sharp, 2004). While some research indicated possible contribution of post-initiation inhibition to translation repression, other studies identified the interruption of mRNA translation at the initiation steps and involvement of specific initiation factors or components (see reviews Gu and Kay, 2010; Wilczynska and Bushell, 2015), highlighting the complexity in studying miRNA-mediated translation inhibition. Meanwhile, processing bodies (P-bodies) and cytoplasmic granules seem associated with mRNA decay and degradation (Sheth and Parker, 2003; Sen and Blau, 2005).

## 4 MicroRNA-controlled posttranscriptional gene regulation of ABC transporters

**FIGURE 5:**
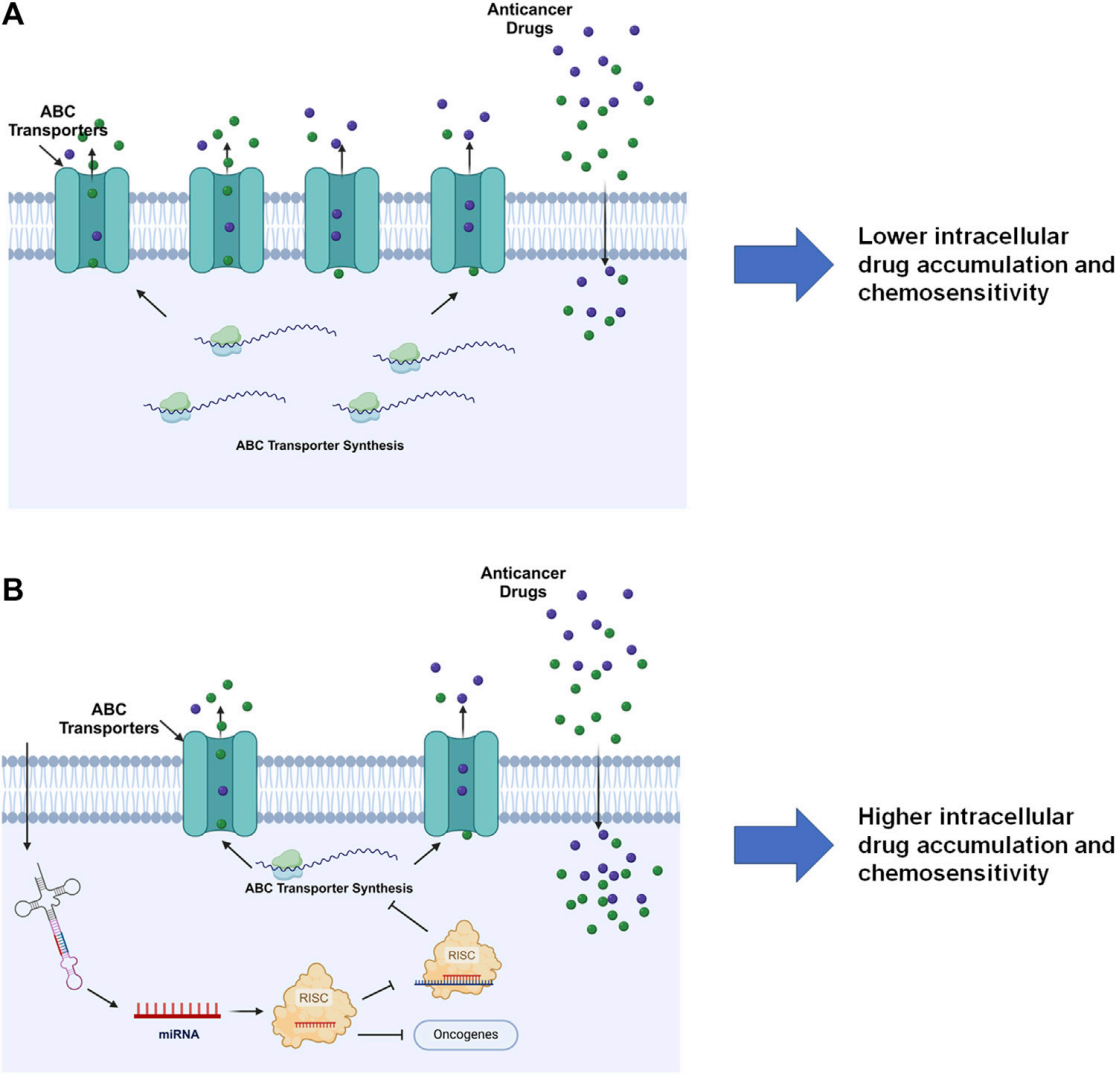
MiRNA-controlled posttranscriptional gene regulation of ABC transporters in drug disposition and chemosensitivity. **(A)** By escaping miRNA mediated PTGR, efflux ABC transporters are overexpressed in cells to pump out substrate drugs, exhibiting lower drug exposure and chemosensitivity. **(B)** Restoration of PTGR of efflux transporters, e.g., through the introduction of bioengineered miRNA molecules, reduces ABC transporter expression, enhances intracellular drug exposure, and increases cell sensitivity to the drugs. Note that miRNAs introduced into the cells may inhibit the expression of some oncogenes to exert anticancer effects. This figure was made by using BioRender.com.

The miRNA mediated PTGR has been established as a critical regulatory mechanism behind target protein outcomes and the whole proteome critical for almost all cellular processes, including ABC transporters vital in ADME and MDR (Figure 5) (Yu, 2009; Yu and Pan, 2012; Haenisch et al., 2014; Yu et al., 2016). Many miRNAs have been shown to impact protein expression of specific ABC transporters through direct targeting of the transporter mRNAs or indirectly acting on their regulators. The reduction of efflux ABC transporter protein levels may subsequently alter the disposition of relevant substrates, such as antineoplastic drugs, and subsequently influence chemosensitivity (Table 2) (Figure 5).

**TABLE 2:** Roles of specific miRNAs in the regulation of human ABC transporter gene expression and influence on drug disposition.

Transporters	miRNAs	Role in ABC transporter expression	Models	References
ABCB1	miR-27a-3p	↑ABCB1	Human ovarian cancer cell line A2780	Zhu et al. (2008)
		↓ABCB1 ↓doxorubicin resistance	Human leukemia cell line K562	Feng et al. (2011)
		↓ABCB1 (via frizzled class receptor 7) ↓5-FU resistance	Human HCC cell line BEL-7402	Chen et al. (2013)
	miR-34a-5p	↓ABCB1 (may relate to the p53) ↓doxorubicin resistance	Human HCC cell line HepG2	Zheng et al. (2019)
	miR-137-3p	↓ABCB1 by targeting YB-1	Human breast cancer cell line MCF-7	Zhu et al. (2013)
	miR-145-5p	↓ABCB1	Human colon carcinoma cell line Caco-2	Ikemura et al. (2013)
	miR-186-5p	↓ABCB1 ↓paclitaxel and cisplatin resistance	Human ovarian cancer cell line A2780	Sun et al. (2015)
	miR-223-3p	↓ABCB1	HCC cell lines Hep3B, HepG2, Huh7, SMMC-7721	Yang et al. (2013)
	miR-298-5p	↓ABCB1 ↓doxorubicin resistance	Human breast cancer cell line MDA-MB-231	Bao et al. (2012)
		↓ABCB1 ↓AEDs resistance	Drug-resistant human brain microvascular endothelial cells (HBMEC/PHT) and astrocytes (U87-MG/DOX)	Xie et al. (2018)
	miR-331-5p	↓ABCB1 ↓doxorubicin resistance	K562 human leukemia cell line	Feng et al. (2011)
	miR-375-3p	↓ABCB1 (via astrocyte elevated gene-1 (AEG-1)) ↓doxorubicin resistance	HCC cell lines Hep3B, HepG2, Huh7	Yoo et al. (2010) He et al. (2012) Fan et al. (2017)
	miR-451-5p	↑ABCB1	Ovarian cancer cell line A2780	Zhu et al. (2008)
		↓ABCB1 ↓doxorubicin resistance	Human breast cancer cell line MCF-7	Kovalchuk et al. (2008)
	miR-491-3p	↓ABCB1 ↓doxorubicin resistance	HCC cell lines Hep3B, SMMC-7721	Zhao et al. (2017)
ABCC1	miR-133a-3p	↓ABCC1 ↓doxorubicin resistance	HCC cell line HepG2	Ma et al. (2015)
	miR-145-5p	↓ABCC1 ↓doxorubicin resistance	Human breast cancer cell line MCF-7	Gao et al. (2016)
	miR-199a-5p	↓ABCC1	Hepatocellular carcinoma	Borel et al. (2012)
	miR-199b-5p			
	miR-296-5p	↓ABCC1	Hepatocellular carcinoma	Borel et al. (2012)
	miR-326-3p	↓ABCC1 ↓doxorubicin resistance	HCC cell line HepG2	Ma et al. (2015)
		↓ABCC1 ↓etoposide (VP-16), doxorubicin resistance	Human breast cancer cell line MCF-7	Liang et al. (2010)
	miR-1291-5p	↓ABCC1 ↓doxorubicin resistance	Human pancreatic cancer PANC-1 cells	Pan et al. (2013)
ABCC2	miR-297-5p	↓ABCC2 ↓oxaliplatin, vincristine, and doxorubicin resistance	Human colon carcinoma cell line HCT-8, HCT-116	Xu et al. (2012a)
	miR-379-5p	↓ABCC2	HCC cell line HepG2	Haenisch et al. (2011)
ABCC3	miR-181b-2-3p	↓ABCC3 ↓doxorubicin resistance	Human breast cancer cell line MDA-MB-231	Zeng et al. (2020)
ABCC4	miR-124a-3p	↓ABCC4	HEK293, and human kidney samples	Markova and Kroetz (2014)
	miR-125a-5p	↓ABCC4	Hepatocellular carcinoma	Borel et al. (2012)
	miR-125b-5p			
	miR-506-3p	↓ABCC4	HEK293, and human kidney samples	Markova and Kroetz (2014)
ABCC5	miR-101-3p	↓ABCC5	Hepatocellular carcinoma	Borel et al. (2012)
	miR-125a-5p			
	Let-7a-5p			
ABCC10	Let-7a-5p	↓ABCC10	Hepatocellular carcinoma	Borel et al. (2012)
	Let-7e-5p			
ABCG2	miR-34a-5p	↓ABCG2 (via Delta-Like 1 (DLL1)) ↓5-FU resistance	Colon cancer side population (SP) cells	Xie et al. (2020)
		↓ABCG2 (via ornithine decarboxylase antizyme 2 (OAZ2)) ↓oxaliplatin resistance	CRC cell line HCT-8	Li et al. (2018)
	miR-212-3p	↓ABCG2 miR-212/ABCG2 is associated with imatinib resistance	Human immortalized myelogenous leukemia cell line K-562	Kaehler et al. (2017); Turrini et al. (2012)
	miR-302a/b/c/d-3p	↓ABCG2 ↓mitoxantrone resistance	Human breast cancer cell line MCF-7	Wang et al. (2016)
	miR-328-3p	↓ABCG2 ↓mitoxantrone resistance	Human breast cancer cell line MCF-7	Pan et al. (2009)
		↓ABCG2 Affects the side population (SP) phenotype	Human CRC cell lines SW1116, LoVo, HCT116, SW480	Xu et al. (2012b)
	miR-519c-3p	↓ABCG2 ↓mitoxantrone resistance	Human breast cancer MCF-7	Li et al. (2011)
	miR-520h-3p	↓ABCG2 Inhibits the cell migration and invasion	Human pancreatic cancer cell line PANC-1	Wang et al. (2010); Li et al. (2011)

### 4.1 ABCB1

MiR-451 represents one of the first identified miRNAs involved in PTGR of ABCB1 (Kovalchuk et al., 2008) (Table 2). Deregulation of miR-451 was revealed in the DOX-resistant, human breast cancer cell line MCF-7/DOX, in which overexpression of ABCB1 contributes to DOX resistance. Luciferase reporter assay showed that miR-451 reduced ABCB1 3′UTR-luciferase activity, validating direct interaction between miR-451 and ABCB1 3′UTR. Further, transfection with miR-451 reduced the protein expression of ABCB1 in MCF-7/DOX cells and increased the sensitivity to DOX (Kovalchuk et al., 2008), suggesting the contribution of miR-451 mediated PTGR of efflux transporter ABCB1 behind MDR.

Likewise, decrease of miR-27a and miR-331-5p were found to be associated with DOX resistance in leukemia cells, and overexpression of these miRNAs increased the sensitivity of these cells to DOX (Feng et al., 2011) (Table 2). Luciferase reporter assay was also used to validate the miR-27a and miR-331-5p binding sites with ABCB1 3′UTR (Feng et al., 2011). In a separate study, miR-27a was showed to modulate ABCB1 expression in human hepatocellular carcinoma (HCC) cells through interference with upstream regulator, and downregulation of ABCB1 increased cell sensitivity to 5-FU (Chen et al., 2013). These findings suggest the potential role of miR-27a in reversing MDR by the reduction of efflux transporter expression. However, opposite effects were reported for miR-27a in human ovarian cancer A2780 cells where miR-27a mimics upregulated ABCB1 expression and increased chemoresistance (Zhu et al., 2008) (Table 2).

The adriamycin (ADM)-selected breast cancer MCF-7/ADM cell line exhibited higher levels of ABCB1 and Y-box binding protein-1 (YB-1), and transfection of miR-137 reduced both YB-1 and ABCB1 levels (Zhu et al., 2013) (Table 2). Further study indicated that miR-137 interfered with YB-1 mRNA, thereby inhibiting protein translation. Notably, YB-1 is a transcription factor that activates ABCB1 expression (Bargou et al., 1997), suggesting the contribution of YB-1 reduction to the suppression of ABCB1 expression in MCF-7/ADM cells by miR-137. Consequently, introduction of miR-137 elevated the sensitivity of MCF-7/ADM cells to anti-cancer drugs DOX, vincristine, and paclitaxel, as manifested by lower IC50 values (Zhu et al., 2013).

Recent study on HCC cells showed that resistance to DOX was correlated with overexpression of ABCB1, whereas miR-491-3p levels were reduced (Zhao et al., 2017) (Table 2). In line with the finding on the transcription factor Sp3 in transcriptional regulation of ABCB1 (Gromnicova et al., 2012), Zhao et al. (2017) demonstrated the actions of miR-491-3p on the 3′UTR of ABCB1 and Sp3 mRNAs that explained the suppression of ABCB1 expression and increase of cell sensitivity to DOX by miR-491-3p.

As ABCB1 transports many anti-epilepsy drugs (AEDs) across the blood-brain barrier (BBB) that may alter the exposure and efficacy of AEDs in patients with refractory epilepsy, another recent study investigated potential use of ABCB1 regulatory miRNA to reverse ABCB1-mediated MDR to AEDs (Xie et al., 2018). Lower levels of miR-298-5p were identified in drug-selected human brain microvascular endothelial cell lines (HBMEC/PHT) and astrocytes (U87-MG/DOX) cell lines, and forced expression of miR-298-5p reduced ABCB1 mRNA and protein levels and consequently, increased the intracellular accumulation of AEDs in drug-resistant HBMEC/PHT and U87-MG/DOX cells (Xie et al., 2018) (Table 2), illustrating the utility of miRNAs to overcome MDR in refractory epilepsy.

### 4.2 ABCC subfamily transporters

ABCC1 expression was found to be elevated in the etoposide-resistant breast cancer cell line MCF-7/VP, accompanied by a pronounced decline in miR-326 levels (Liang et al., 2010) (Table 2). Following the identification and validation of miR-326 binding site within ABCC1 3′UTR, transfection of MCF-7/VP cells with miR-326 was showed to reduce ABCC1 mRNA and protein levels as well as sensitivity to etoposide and DOX (Liang et al., 2010). In another study, miR-1291-5p was revealed to be derived from the small nucleolar RNA H/ACA box 34 (SNORA34) in human pancreatic carcinoma PANC-1 cells overexpressing ABCC1 (Pan et al., 2013). Control of ABCC1 protein outcomes by miR-1291 was verified by both gain- and loss-of-miR-1291 function approaches, besides the validation of miR-1291 response elements within ABCC1 3′UTR. As miR-1291 did not affect ABCC1 mRNA stability, PTGR of ABCC1 by miR-1291 is likely attributable to translation repression mechanism. In addition, downregulation of ABCC1 by miR-1291 elevated intracellular DOX accumulation and sensitized PANC-1 cells to DOX (Pan et al., 2013) (Table 2), illustrating the importance of miRNA-controlled PTGR in drug disposition and MDR.

In the multidrug-resistant, human colorectal carcinoma (CRC) cell line HCT-116/L-OHP overexpressing efflux transporter ABCC2, miR-297 level was found to be reduced (Xu K. et al., 2012) (Table 2). Reporter assay revealed that miR-297 suppressed ABCC2 3′UTR-luciferase activity. As miR-297 was effective to repress ABCC2 expression, overexpression of miR-297 in drug-resistant CRC cell lines sensitized the cells to anticancer drugs, including oxaliplatin, vincristine, and DOX, which was associated with an enhanced apoptosis of multidrug-resistant CRC cells compared to antineoplastic drug monotherapy (Xu K. et al., 2012).

One most recent study (Zeng et al., 2020) first showed that curcumol improved the sensitivity of drug-resistant, triple-negative breast cancer (TNBC) MDA-MB-231 cells to DOX *in vitro* and *in vivo*. That was related to the increase of miR-181b-2-3p levels which was found to target ABCC3 directly. Furthermore, curcumol activated a transcription factor, NFAT1, which could directly bind to the promoter region of miR-181b-2-3p to enhance its expression. Therefore, this NFAT1/miR-181b-2-3p/ABCC3 axis was likely involved in the sensitization of TNBC cells to DOX by curcumol (Table 2).

There are also many other reports on PTGR of human ABCC transporters by specific miRNAs, such as ABCC4 by miR-124 and -506 (Markova and Kroetz, 2014), as well as ABCC5 and ABCC10 by let-7 (Borel et al., 2012), as summarized in Table 2. Likewise, repression of ABCC transporter protein levels by the miRNAs could be translated into an altered drug exposure and response in the cells. Together, these findings not only demonstrate the involvement of miRNA-controlled PTGR in variations in efflux ABCC transporter expression and chemosensitivity but also provide insights into development of new remedies to overcome MDR (Choi and Yu, 2014; Yu et al., 2016).

### 4.3 ABCG2

Consisting of miR-519c binding sites within the 3′UTR, ABCG2 was showed to be repressed by miR-519c in the S1 colon cancer cells (To et al., 2008) (Table 2). However, the drug-resistant subline S1MI80 with truncated ABCG2 3′UTR lacking miR-519c binding sites escaped miR-519c mediated PTGR, providing new insights into ABCG2 overexpression conferring MDR in these cells (To et al., 2008). In the mitoxantrone-resistant breast cancer cell line MCF-7/MX100 overexpressing ABCG2 transporter, miR-328 was able to increase the sensitivity to mitoxantrone by decreasing the ABCG2 protein expression via complementary targeting the 3′UTR of ABCG2 mRNA (Pan et al., 2009). Further, miR-520h was found to modulate ABCG2 expression in PANC-1 cells and contribute to cancer cell migration and invasion (Wang et al., 2010). Another side-by-side study on the efficiency of miR-328, -519c, and -520h revealed that miR-328 and -519c were effective to repress ABCG2 protein levels in MCF-7/MX100 cells and thus, increase intracellular drug accumulation and chemosensitivity (Li et al., 2011). By contrast, miR-520h was found to be ineffective to regulate ABCG2 expression in MCF-7/MX100 cells in which miR-520h levels were unchanged, highlighting the importance to monitor miRNA levels in these studies.

Some recent studies demonstrated the regulation of ABCG2 by several other miRNAs, including miR-302a/b/c/d in mitoxantrone-resistant breast cancer cells (Wang et al., 2016) and miR-212 in imatinib-resistant leukemia cells (Kaehler et al., 2017) (Table 2). Both studies also showed the effectiveness of such miRNAs to sensitize drug-resistant cancer cells, supporting the roles of miRNA-controlled PTGR of the efflux ABC transporter as well as implications to drug disposition and MDR.

## 5 Recombinant RNAs to study ABC transporter posttranscriptional gene regulation

Recombinant DNA (rDNA) technology, which modifies genetic materials to produce specific products or introduce certain traits into living organisms, has been widely used for precise molecular, cellular, or systems studies on ADME genes (Cronin and Yu, 2023). The basis of rDNA technology is the manipulation of DNA sequences within host organisms using viable vectors or plasmids (Vieira and Messing, 1982). Application of rDNA technologies enables the production of gene products related to drug metabolism and disposition, such as drug-metabolizing enzymes (Dai et al., 1993; Crespi and Miller, 1999; Gonzalez, 2004) and efflux transporters (Galluccio et al., 2022; Cronin and Yu, 2023), which have greatly improved our understanding of roles of ADME genes in drug metabolism and disposition as well as possible influence on therapy.

While heterologous overexpression of RNAs proved to be challenging, novel technologies have been developed to achieve high yield production of recombinant miRNAs (named BERAs or BioRNAs) for PTGR studies, by using unique transfer RNA (tRNA) fused pre-miRNA carriers (Yu et al., 2019; Cronin and Yu, 2023; Traber and Yu, 2023). The tRNA fused pre-miRNA (e.g., pre-miR-34a) carriers allow a consistent, high-level and large-scale heterologous overexpression (e.g., >30% of total bacterial RNA) as well as high-yield production (e.g., tens milligrams from 1 L fermentation) of high-purity, target BioRNAs (e.g., >97% homogeneity, by HPLC; <3.0 EU/µg RNA) bearing target miRNAs, siRNAs, aptamers, or other forms of small RNAs (Chen et al., 2015; Ho et al., 2018; Li et al., 2021; Traber et al., 2024), in contrast to the tRNA scaffold (Ponchon et al., 2009) that commonly leads to the absence of heterologous expression of target RNAs or merely offers relatively a low yield (Li et al., 2014; Chen et al., 2015). Distinguished from conventional miRNA reagents synthesized *in vitro* through chemical or enzymatic reactions consisting of either extensive artificial modifications or none, BERAs or BioRNAs are similar as natural RNAs made and folded *in vivo* and being comprised of only a few essential posttranscriptional modifications (Li et al., 2015; Wang et al., 2015).

Recombinant BioRNA/miRNAs are precisely processed to target miRNAs in human cells to selectively regulate target gene expression, including many human ABC transporters (Table 3; Figure 5). For instance, recombinant miRNA let-7c-5p was successfully produced and introduced into human HCC Huh7 cells, leading to a sharp increase of let-7c-5p levels (Jilek et al., 2020). This resulted in a significant downregulation of ABCC5 protein levels in Huh7 cells, while ABCC4 and ABCG2 levels were not or minimally affected (Table 3). Additionally, coadministration of BioRNA/miR-7c-5p and 5-FU showed a strong synergism to inhibit HCC cell proliferation, partially attributable to the elevation in intracellular accumulation of 5-FU, a known substrate of ABCC5 transporter (Wijnholds et al., 2000; Pratt et al., 2005) (Table 1). As another example, Li et al. (2019) produced BERA/miR-328-3p that was revealed to control PTGR of ABCG2 transporter overexpressed to render mitoxantrone drug resistance in human breast cancer cells, namely MCF-7/MX100 cells (Pan et al., 2009; Li et al., 2011) (Table 3). After transfection with the BERA/miR-328-3p, the levels of miR-328-3p were greatly elevated in MCF-7/MX100 cells, leading to the reduction of ABCG2 and consequently, greater cellular exposure of and sensitivity to MX (Li et al., 2019). Rather, treatment of human placental BeWo cells with BERA/miR-328-3p did not alter intrinsic ABCG2 levels, intracellular MX accumulation, and chemosensitivity, indicating the difference in PTGR mechanism of ABCG2 between BeWo and drug resistant MCF-7/MX100 cells.

**TABLE 3:** Utilization of novel recombinant miRNA agents in studying transporter gene expression and impact on xenobiotic flux and multidrug resistance. GLUT1/*SLC2A1*, glucose transporter protein type 1, gene symbol solute carrier family two member 1; LAT1/*SLC7A5*, large neutral amino acid transporter 1, gene symbol solute carrier family seven member 5.

Bioengineered miRNAs	Targets	Findings	Model systems	References
miR-1291-5p	ABCC1	miR-1291 reduces ABCC1 transporter protein levels and improves cell sensitivity to doxorubicin	PANC-1 and MCF-7 cells	Li et al. (2015)
	GLUT1	miR-1291 downregulates GLUT1 to suppress glucose uptake which alters glycolysis and improves cisplatin efficacy	Human AsPC-1 and PANC-1 cells	Tu et al. (2020)
miR-124-3p	ABCC4	miR-124 reduces the ABCC4 protein expression contributing to the inhibition of cancer cell proliferation and metastasis	A549 cells	Ho et al. (2018)
miR-328-3p	ABCG2	miR-328 downregulates ABCG2 expression and sensitizes drug-resistant cells to m itoxantrone	MCF-7/MX100 cells	Li et al. (2019)
	GLUT1/*SLC2A1*	miR-328 decreases the protein levels of GLUT1 and LAT1 associated with its synergistic antiproliferation activity with doxorubicin or cisplatin	Human MG63 and 143B cells	Yi et al. (2020)
	LAT1/*SLC7A5*			
let-7c-5p	ABCC5	let-7c downregulates the ABCC5 protein expression and increases the intracellular 5-FU accumulation	Huh7 cells	Jilek et al. (2020)

Besides efflux ABC transporter, bioengineered miRNAs have been utilized to investigate PTGR of some solute carrier transporters (Tu et al., 2020; Yi et al., 2020) and enzymes (Li et al., 2014; Li et al., 2019; Tu et al., 2020) in ADME (Table 3). The involvement of miR-27b-3p in PTGR of human cytochrome P450 3A4 (CYP3A4) was demonstrated not only by a tRNA fused pre-miR-27b (Li et al., 2014) but also the tRNA/pre-miR-34a carrier based miR-27b-3p (Li et al., 2019). Following the downregulation of CYP3A4 proteins by recombinant miR-27b-3p, cellular midazolam 1′-hydroxylation capacity was reduced, indicating the involvement of miR-27b-3p signaling in drug metabolism. Further, miR-1291-5p was selectively released from recombinant miR-1291 in human pancreatic cancer cells (Tu et al., 2020). The suppression of arginosuccinate synthase (ASS1) in L3.3 cells by miR-1291-5p altered arginine homeostasis and increased the sensitivity of cells to arginine deprivation therapy (Tu et al., 2020). On the other hand, the reduction of glucose transporter protein type 1 (GLUT1) by miR-1291-5p decreased glucose uptake and glycolysis capacity and improved cisplatin efficacy in human AsPC-1 and PANC-1 cells (Tu et al., 2020).

Research on PTGR in ADME with recombinant RNAi molecules has advanced our knowledge of complex mechanisms behind interindividual variations in ADME and offered clues to developing new therapies. Indeed, a total of 6 RNAi therapeutics have been approved by the United States Food and Drug Administration for the treatment of various disorders, such as patisiran for hereditary transthyretin-mediated amyloidosis and nedosiran for primary hyperoxaluria type 1, respectively (Yu et al., 2019; Yu et al., 2020; Traber and Yu, 2023, 2024). Many studies have demonstrated the efficacy and safety of recombinant miRNAs and siRNAs in the control of tumor growth in clinically relevant animal models (Ho et al., 2018; Jilek et al., 2019; Tu et al., 2019; Deng et al., 2021; Ning et al., 2022; Chen et al., 2023; Luo et al., 2024) that are amenable to clinical investigations.

## 6 Conclusion and perspectives

The ubiquitous expression of membrane ABC transporters in human body maintains normal biologic processes through preserving endobiotic homeostasis, protecting against environmental toxins, and limiting the exposure to other xenobiotics. ABC transporters localized among metabolic organs pose an important role in substrate drug absorption, distribution, excretion, and overall PK properties, which may be altered among patients with certain conditions. Intrinsic or acquired overexpression of ABC transporters in the organisms or diseased cells has been shown to confer MDR by reducing drug exposure, which remains a major challenge in treating infectious diseases and cancers. While targeting efflux ABC transporters is a potential strategy to overcome MDR, clinical trials on previous generations of inhibitors reveals just a limited benefit whereas some toxicity. Therefore, a thorough understanding of complex mechanisms underlying dysregulation of ABC transporters would offer new insights into developing other possible means to improve therapies.

With the discovery of functional miRNAs derived from the genome, many recent studies have demonstrated the contribution of specific miRNAs in PTGR of various ABC transporters by targeting respective transcripts. Coadministration of certain miRNAs is proven effective to sensitize drug-resistant cells or tumors to anticancer drugs through the reduction of efflux ABC transporter expression, which rather warrants more extensive studies and clinical investigations. Furthermore, novel, *in vivo* fermentation based RNA molecular bioengineering technologies have been developed to offer high-quality, recombinant miRNA molecules that are a unique addition to conventional miRNA agents synthesized *in vitro* chemically or enzymatically. Bioengineered miRNA molecules have been revealed functional to modulate the expression of ABC transporters and other ADME genes and subsequently alter cellular drug metabolism and transport capacities as well as chemosensitivities. Such recombinant miRNA and RNAi molecules represent a novel class of tools for ADME and broader general biomedical research while holding promise as therapeutics.
